# Development of a fast and precise potency test for BCG vaccine viability using flow cytometry compared to MTT and colony-forming unit assays

**DOI:** 10.1038/s41598-023-38657-x

**Published:** 2023-07-18

**Authors:** Hend M. Moghawry, Mohamed E. Rashed, Kareeman Gomaa, Sameh AbdelGhani, Tarek Dishisha

**Affiliations:** 1 Department of Pharmaceutical Microbiology and Immunology, Faculty of Pharmacy, Beni-Suef University, Beni-Suef, 625 11 Egypt; 2 General Administration of Biological Products, Central Administration of Biological and Innovative Products and Clinical Trials, Egyptian Drug Authority (EDA), Giza, Egypt; 3 Clinical and Chemical Pathology Department, Faculty of Medicine - Kasr Al-Ainy, Cairo University, Cairo, Egypt; 4 Department of Pharmacy, Jewish Hospital, University of Louisville, Louisville, KY 402 02 USA

**Keywords:** Live attenuated vaccines, Assay systems, Biochemical assays, Flow cytometry

## Abstract

In a precarious world of rapidly growing pandemics, the field of vaccine production has witnessed considerable growth. Bacillus Calmette-Guérin (BCG) is a live-attenuated vaccine and a part of the immunization program in 157 countries. The quality control is based on a potency test through viable cell enumeration. The colony-forming unit (CFU) assay is the official method, however, it often yields fluctuating results, suffers from medium cracking, and requires lengthy analysis (~ 28 days). Flow cytometric analysis was proposed earlier, but it was coupled with a Coulter counter for measuring the entire bacterial population (live/dead). In the present study, thiazole orange/propidium iodide dyes supplemented with fluorogenic reference beads were employed for viable counting, eliminating the need for a Coulter counter. Both the flow cytometry and the colorimetric technique employing tetrazolium salt were validated and compared to the CFU assay. The colorimetric assay displayed high precision, accuracy, and a strong positive correlation with the CFU assay. The flow cytometry assay demonstrated high precision and a notable ability to distinguish different forms of BCG cells (live, injured, and dead). It also exhibited a perfect positive correlation with the CFU assay. Both methods reduced the analysis time by > 26 days and eliminated the need for human intervention by automating the test.

## Introduction

Tuberculosis (TB) is a contagious disease that originated about 150,000 years ago^1^. It is caused by a slow-growing microorganism known as *Mycobacterium tuberculosis*, which can affect either the lungs causing pulmonary TB, or other tissues causing extra-pulmonary TB^2–4^. TB is considered one of the top ten causes of mortality worldwide, with approximately ten million people infected and about 1.5 million deaths annually^5^.

At the Pasteur Institute, Calmette and Guérin developed the Bacillus Calmette-Guérin (BCG) vaccine against the TB using *Mycobacterium bovis* strain isolated from an infected cow with tuberculous mastitis. The strain was sub-cultured several times over a period of 13 years, resulting in the live-attenuated bacilli^6^. Nowadays, the BCG vaccine plays a leading role in preventing TB and has contributed to the immunization of more than 3 billion people worldwide^7^. Moreover, it was evaluated as intravesical immunotherapy for the prevention of recurrent superficial bladder tumors in 1976^8^, and later on, was approved by the Food and Drug Administration (FDA) in 1990^9^.

Quality control of vaccines is a fundamental parameter in ensuring the safety, efficacy, and quality of manufactured products. Since the BCG vaccine is derived from live-attenuated bacteria, the potency test is a crucial quality parameter. Currently, the colony-forming unit (CFU) assay is the official golden method recommended by the World Health Organization (WHO) and recognized by European Pharmacopeia (EP)^10,11^. It depends on counting the number of viable cells of *M. bovis* after cultivation on Lowenstein-Jensen (LJ) media and incubation at 37 °C for around 4 weeks. However, it suffers from cracking of the culture media as a result of dryness (Supplementary Fig. S1A,B), and clumping of mycobacterial colonies, besides being labor-intensive, time-consuming, and highly subjective to personal error^12–14^.

Various automated methods have been developed for the analysis of BCG vaccines which allow rapid detection of viable cells and improve the quality control and standardization procedures. For instance, the colorimetric tetrazolium salt assay depends on the redox reactions of tetrazolium salts, by the action of metabolic enzymes of active living cells, yielding a purple formazan precipitate. The quantity of the colored precipitate is directly proportional to the number of viable cells and can be easily detected spectrophotometrically^15–17^. The ATP luminescence assay measures the bioluminescence resulting from the reaction of firefly luciferase with intracellular ATP of only living cells^18^. However, these methods have poor correlation with the official CFU assay and require additional processing steps, such as extraction of the intracellular ATP and dissolving the formazan precipitate, among others^15,18,19^.

Recently, flow cytometry assays were developed based on the use of highly sensitive laser-based flow cytometer technology for cell counting^14,20^. In this case, two strategies can be employed, known as dye-exclusion and dye-uptake^21^. The dye-exclusion method depends on the ability of only living cells to exclude fluorescent dyes like propidium iodide (PI) mediated by their intact cell membranes. However, dead cells having permeable membranes can easily absorb these dyes. On the other hand, the dye-uptake concept refers to the ability of only viable cells to uptake certain stains like fluorescein diacetate (FD)^21^.

Yang et al.^14^ employed the dye-exclusion strategy using PI for determining the percentage of dead cells in the BCG vaccine^14^. On the other hand, Gweon et al.^20^ used the dye-uptake strategy for direct staining of living cells with FD for measuring the percentage of living cells^20^. In both cases, the flow cytometer was coupled to a Coulter counter for counting the total number of cells (living and dead); multiplying this number by the percentage of living/dead cells yields the absolute number of the corresponding cells^14,20^. However, these studies require the use of multiple apparatus making it liable to handling variations. Moreover, the correlation of these assays with CFU assay was variable and ranged from 0.7 to 0.95^14,20^.

Adapting reference fluorogenic beads with a known quantity during the flow cytometry assays represents a rapid quantitative parameter for better identification, sorting, and quantification of various types of cells in a short time using a single apparatus with minimal human efforts^22–26^.

In the present study, the pharmacopeia-recommended CFU assay was compared to both the colorimetric and the flow cytometry assays regarding BCG vaccine potency. Sauton’s solution supplemented with 10% Tween 80 was evaluated for reconstituting the lyophilized BCG, which is more cost-efficient than Middlebrook 7H9 broth. Additionally, thiazole orange/ propidium iodide (TO/PI) dyes of the cell viability kit supplemented with reference fluorogenic beads were used for viable counting of the BCG vaccine instead of the Coulter counter. Finally, both methods were subjected to the assessment of validation parameters, including intra- and inter-assay precision, accuracy, and correlation with the CFU assay, among others whenever applicable.

## Results

### Validation and verification of colorimetric MTT assay

#### Calibration curve of colorimetric MTT assay and linearity

**Figure 1:**
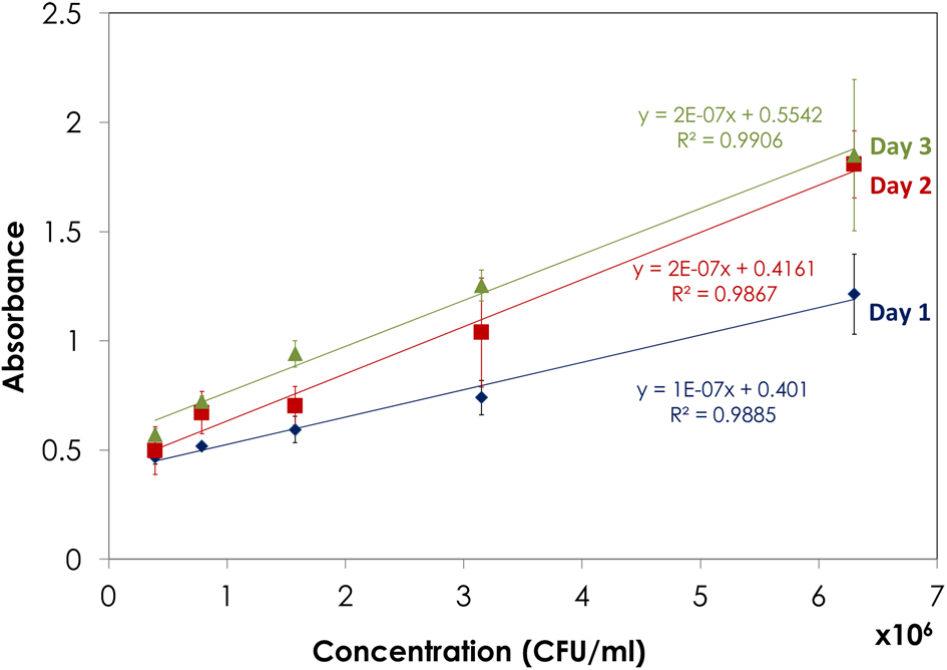
Calibration curves of standard BCG vaccine using the colorimetric MTT assay performed during three consecutive days; (filled diamond) Day 1, (filled square) Day 2 and (filled triangle) Day 3.

The International Council for Harmonization of Technical Requirements for Pharmaceuticals for Human Use (ICH) has provided guidelines in its Q2 document for the validation of analytical procedures. To obtain the calibration curve, five standard BCG concentrations were used within the range of 0.39 × 10^6^ to 6.30 × 10^6^ CFU/ml. Each concentration was analyzed in triplicate (Fig. 1). The resulting absorbance at 570 nm demonstrated a positive linear relationship with viable cell concentration with a slope of the regression line equal to 1.00 × 10^–7^, a y-intercept of 0.40, an R^2^ of 98.85%, and a Pearson correlation coefficient (r) of 0.99. The calibration curve equation was applied to estimate the viable cell concentration (CFU/ml) of the samples in the following steps.

The calibration curve was repeated on two consecutive days, and both showed a good positive linear relationship with an R^2^ exceeding 98% (Fig. 1). The means of the slopes and intercepts of the three calibration curves were not significantly different at P < 0.05.

#### Intra-precision of colorimetric MTT assay

The intra-assay precision (repeatability) is a measure of the variance between data points within an assay^27^. The assay was applied to the results of 10 BCG vaccine batches, each was run in triplicate (Supplementary Table S1). The percentage coefficient of variation (%CV) for each sample was calculated, and the average of the individual %CVs is reported as the intra-assay CV%, which was 6.76%. Generally, an intra-assay %CV of less than 10% is acceptable^27^.

#### Inter-precision of colorimetric MTT assay

The inter-assay precision is a measure of the variance between runs of sample replicates on different plates (days). Therefore, four BCG vaccine batches representing the high, intermediate, and low levels were analyzed for three consecutive days. The mean of each concentration over the three days was calculated and used to calculate the mean of the means, standard deviation (SD) of the means, and %CV of the means (Table 1). The average of the %CV of the means is considered as the inter-assay CV%, which was 12.9%. Generally, an inter-assay CV% below 15% is considered acceptable^27^.

**Table 1:** Inter-assay precision analysis of the colorimetric MTT assay considering the results of 4 reference BCG analyzed in triplicates over three consecutive days.

Stock solution		Estimated BCG viability*			
		6.30 (high)	3.15	1.58	0.79 (low)
Day 1	Read I*	5.32	2.39	1.44	0.891
	Read II*	8.09	3.42	1.13	0.891
	Read III*	5.95	2.31	2.08	1.05
Mean 1*		6.45	2.71	1.55	0.943
Day 2	Read I*	6.69	4.67	1.35	1.68
	Read II*	5.59	3.06	0.93	0.826
	Read III*	6.90	0.90	1.74	1.08
Mean 2*		6.40	2.88	1.34	1.20
Day 3	Read I*	7.92	3.63	1.98	0.80
	Read II*	5.75	3.35	1.51	0.95
	Read III*	4.72	2.97	2.03	0.71
Mean 3*		6.13	3.32	1.84	0.82
Mean of means*		6.33	2.97	1.58	0.99
SD of means*		0.17	0.31	0.25	0.19
%CV of means		2.74	10.58	15.96	19.48
Inter-assay CV% = (2.74 + 10.58 + 15.96 + 19.48)/4 = 12.19%					

*(× 10^6^ CFU/ml).

#### Accuracy of colorimetric MTT assay

Accuracy expresses the closeness of agreement between the value which is accepted and that of a reference value. According to ICH Q2 guidelines, accuracy should be assessed using a minimum of nine determinations over a minimum of three concentration levels covering the specified range^28^. In the present study, five samples with triplicate analysis were employed. The percentage error was calculated for the mean (0.46%); subtracting this from 100% yielded the percentage accuracy (99.54%) (Table 2). This indicates that the colorimetric MTT assay has high accuracy.

**Table 2:** Accuracy of the colorimetric MTT assay for determination of cell viability of known concentrations of reference BCG vaccine.

Batch no.	Expected*	Observed*	% Error
A	6.30	6.33	0.48%
B	3.15	3.13	0.63%
C	1.57	1.63	3.82%
D	0.79	0.83	5.84%
E	0.39	0.33	15.27%
Mean	2.44	2.45	0.46%

*(× 10^6^ CFU/ml).

#### Detection limit (DL) of colorimetric MTT assay

To assess the detection limit, the data from the reference BCG calibration curves obtained over three consecutive days were used. The SD of the response was calculated using the SD of the intercepts of the regression line and the slope as recommended by the ICH guidelines^28^. The LOD was 0.51 ± 0.04 (absorbance units).

#### Correlation between colorimetric MTT assay and official CFU assay

**Figure 2:**
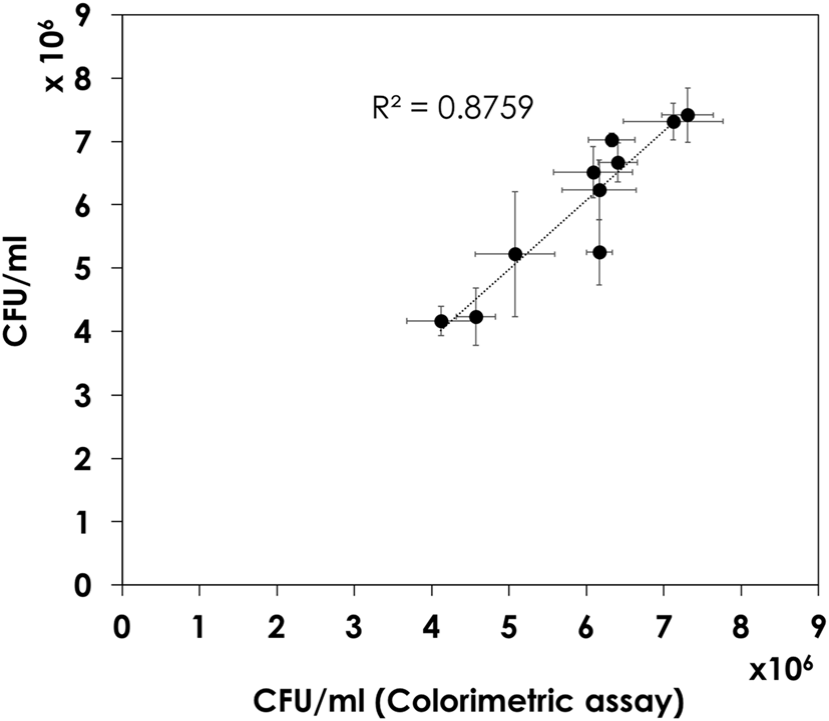
Correlation of the viability of 10 Bacille Calmette- Guérin (BCG) vaccine batches as determined by the official colony-forming unit (CFU) assay and colorimetric MTT assay.

The correlation between the colorimetric MTT assay and the official CFU assay was assessed by measuring the number of viable cells for 10 BCG vaccine batches using both techniques. Supplementary Table S2 shows a comparison of the means of the two analytical procedures. The Pearson correlation coefficient (r) was calculated from the results to be 0.94, indicating a strong positive correlation. Figure 2 represents the correlation behavior between the two methods, where an R^2^ of 0.88 was obtained. The MTT assay preserves a good linearity at lower BCG vaccine concentrations as shown in Supplementary Fig. S3.

A Student’s *t* test was applied to investigate the significance of the mean differences between the two methods. The test indicates no significant differences between the proposed colorimetric and the official method for the determination of the viability of the BCG vaccine.

### Validation and verification of flow cytometry assay

Flow cytometry method validation is aimed at ensuring that the data are credible and reproducible. Therefore, a limited validation protocol is selected to provide minimal recommended parameters for research conditions. These protocols include verification of specificity, intra- and inter-precision parameters, and correlation to the standard CFU assay. The data obtained is regarded as quasi-quantitative due to its numerical nature to the sample itself and not derived from a calibration curve or a reference material. Therefore, accuracy parameter verification is not applicable in this type of analysis. Moreover, sensitivity parameter verification is not required in this assay as needed only in rare events^24,25,29,30^.

#### The ability of the flow cytometer to distinguish live and dead mycobacterial cells (specificity)

**Figure 3:**
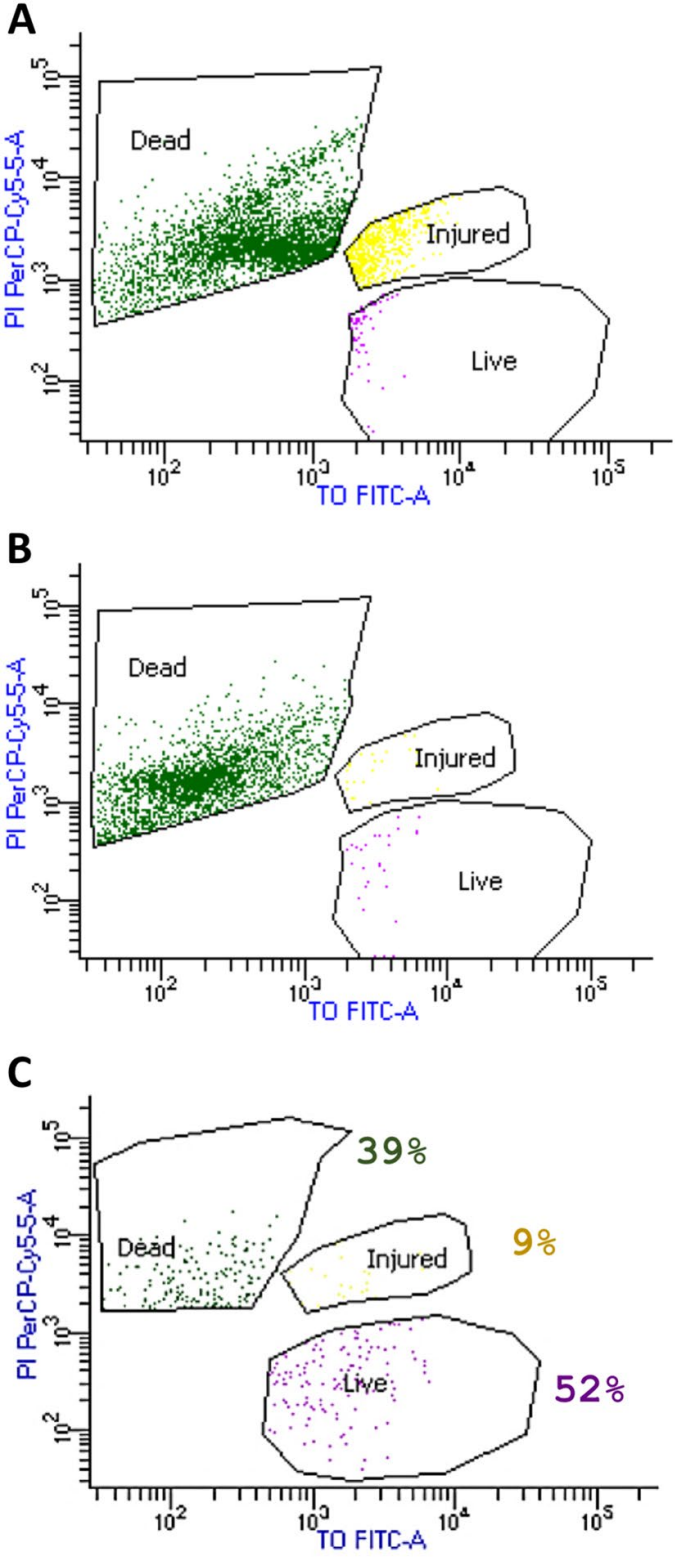
Live/dead discrimination of BCG cells using BD cell viability kit in flow cytometer. The figure represents FL1 vs*.* FL3 dot plots of bacteria stained with TO and PI, gated on combined parameters FSC, SSC, and FL2 ((R1 or R2) and R3). Dead BCG cells (TO^+^PI^+^) were gated on the upper left quadrant zone, which is positive for PI and negative for TO, while, injured (TO^+^PI^intermediate^) and viable (TO^+^PI^–^) BCG cells were seen on the upper and lower right quadrant zones, respectively. (**A**) formalin-treated BCG vaccine by exposure to 2.5% formaldehyde, (**B**) Heat-killed BCG vaccine by autoclaving at 121 °C for 30 min, and (**C**) BCG vaccine sample with 3.47 × 10^6^ CFU/ml. The figure shows the ability of flow cytometry to distinguish between living, injured and dead cells of the BCG vaccine. Data acquisition, data analysis and visualization were done using the FACSDiva software. (*TO* thiazole orange, *PI* propidium iodide).

Cell viability of the reference BCG vaccine was assessed using the BD Cell Viability Kit of TO/ PI dyes. TO is a permanent dye that can enter all cells, either live or dead, allowing simple discrimination from the background noise. While PI dye can only diffuse into dead cells with permeable membrane and intercalate into the double-stranded nucleic acid^31^. To study the ability of the flow cytometer to distinguish between living and dead cells, the BCG vaccine was treated with heat or formalin to kill the viable cells. The CFU assay revealed no colony formation in both cases (Supplementary Fig. S1C). In flow cytometry, dead cells were observed only in the upper left quadrant zone, which is positive for PI and negative for TO (Fig. 3). While injured and viable BCG cells were seen in the upper and lower right quadrant zones, respectively (Fig. 3). Figure 3A and B revealed that heat treatment is less destructive to cells resulting in a higher percentage of injured cells compared to formalin treatment. Only a few numbers of events of live cells were observed in both cases, which were negligible (below 1%) of all detected events.

On the other hand, Fig. 3C represents the BCG vaccine sample with an estimated 3.47 × 10^6^ CFU/ml, showing clear discrimination between live (52%), injured (9%), and dead (39%) cells.

#### Intra-precision of flow cytometry

Precision is one of the most critical parameters for the validation of flow cytometry. Verification of intra-assay precision parameters was applied to 10 BCG vaccine batches. Supplementary Table S3 presents the results of the flow cytometry analysis of the 10 batches, showing the means, SD, and CV% of each sample. The average %CV (intra-assay CV%) was 6.08%, which is within the acceptable range for cell-based assays^32^.

#### Inter-precision of flow cytometry

The inter-assay precision was performed using four samples, each analyzed in triplicates for two days (n = 4, number of determinants = 24) (Table 3). The means of each analysis and concentration were calculated. Finally, the mean of the means, SD of the means, and CV% of the means were calculated. The inter-assay CV% was 6.28%, which is within the acceptable range for cell assays^32^.

**Table 3:** Inter-assay precision analysis of the flow cytometry technique for analysis of 4 BCG vaccine samples over two consecutive days using BD viability kit with thiazole orange (TO)/propidium iodide (PI) dyes and liquid counting beads.

Batch no.	Day	Estimated BCG viability			Parameters		Mean of means*	SD of means*	CV% of means	Inter-assay CV%
		Read 1*	Read 2*	Read 3*	Mean*	SD*				
Sample K	Day 1	4.49	4.19	4.09	4.25	0.21	4.35	0.13	3.08	6.28%
	Day 2	4.65	4.50	4.19	4.44	0.24				
Sample L	Day 1	5.00	4.88	4.69	4.85	0.16	4.43	0.60	13.46	
	Day 2	3.99	4.00	4.04	4.01	0.03				
Sample M	Day 1	4.52	3.85	4.61	4.33	0.41	4.23	0.13	3.15	
	Day 2	4.12	4.42	3.88	4.14	0.27				
Sample N	Day 1	3.67	3.08	3.22	3.32	0.31	3.20	0.17	5.41	
	Day 2	3.04	3.18	3.01	3.08	0.09				

*(× 10^6^ CFU/ml).

#### Correlation between flow cytometry assay and official CFU assay

**Figure 4:**
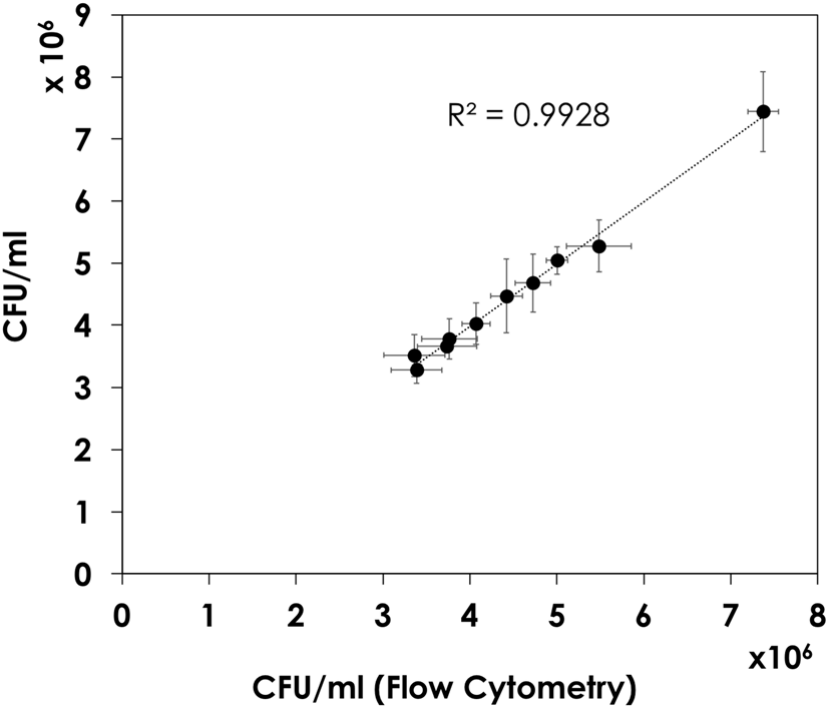
Correlation of the viability of 10 Bacille Calmette- Guérin (BCG) vaccine batches as determined by the official colony-forming unit (CFU) assay and flow cytometry using BD cell viability kit with thiazole orange (TO)/propidium iodide (PI) dyes and liquid counting beads.

To verify the utility of the flow cytometric assay for the analysis of BCG vaccine potency, ten samples were measured by both CFU assay and flow cytometry assay. Supplementary Table S4 shows the estimated titers from the flow cytometric assay compared to that of the official CFU assay. The P-value of the Student’s *t* test was 0.98, indicating no significant difference between viability analysis by both methods. Figure 4 illustrates how the flow cytometric assay correlates to the CFU assay. The Pearson correlation coefficient (r) was 1.00 indicating a perfect positive correlation between the two measurement techniques. The regression line had an R^2^ of 0.99 (Fig. 4).

## Discussion

In the precarious world of rapidly growing pandemics, the field of vaccine production has witnessed considerable growth. The BCG vaccine is a live-attenuated bacterial vaccine that is becoming increasingly important worldwide^4^. It is included in the immunization program of approximately 157 countries worldwide^33^. The vaccine enhances neonates' innate immune system, saving their lives from the rapidly progressive TB disease^34,35^. Recently, it was reported to be more effective than the chemical counterpart, Epirubicin, with regards to preventing recurrent transitional cell carcinoma of the bladder^36^.

In the downstream processing of the vaccines, quality control and lot release are the final steps. Viable cell enumeration is a crucial parameter in the assessment of the live-attenuated bacterial vaccine potency^4^. The official golden method for viability analysis is the CFU assay. However, this method is highly subjective. In 2017, Gweon et al. demonstrated that the CV% between different laboratories can be as high as 95% (with an average of 49%) when using CFU assay for analysis^20^.

Various enhanced biochemical approaches were developed for determining the potency of the BCG vaccine. Once appropriate method validation and standardization have been performed, these approaches could be approved by the European Pharmacopoeia (EP). In the present study, the colorimetric technique using MTT salt and the flow cytometry technique employing the cell viability kit of TO/PI fluorescent dyes supplemented with reference counting beads were employed to measure the BCG vaccine potency. Both approaches are automated, minimizing the need for human intervention. They have also shortened the analysis time considerably from 28 days in the case of CFU assay; spent mainly for the cultivation of the mycobacterial cells, to only 1 or 2 days for the flow cytometry and colorimetric assay, respectively (corresponding to 96.43% and 92.86% reduction in analysis time). Furthermore, an earlier study demonstrated that the flow cytometry potency assay can be completed in just 4 h (99.40% reduction in time)^14^.

Although the colorimetric MTT assay was investigated earlier for the BCG vaccine potency test, the study was more oriented towards optimization of the assay components where Sauton’s solution was used for diluting and reconstituting the BCG vaccine rather than the expensive Middlebrook 7H9 broth. Verification of the validation parameters of the colorimetric MTT assay revealed that the method was sensitive to viable cell concentration where linear calibration curves were obtained within the tested range. The observed variation in the standard curves can be attributed to the biological nature of the BCG vaccine, as well as the MTT assay’s reliance on measuring metabolic activity as an indicator of cell viability. Consequently, repeating the same test multiple times will not yield identical results. Therefore, generally guidelines provide a range of acceptable values rather than a specific value.

The MTT assay was precise and accurate, with intra- and inter-assay CV% within the acceptable ranges. Furthermore, it showed a high correlation with the CFU assay as confirmed by Pearson’s Correlation Coefficient. Although the LOD was slightly high (0.51 absorbance units), the assay’s sensitivity to lower concentrations can be improved by optimizing various parameters, such as the incubation time^15,16^ and the composition of the extraction solvent^15,37^.

In the case of flow cytometric analysis, combining the TO and PI dyes for staining the whole population and dead cells, respectively, yielded data that are superior to that obtained earlier using only one staining dye. TO dye stains all the forms of BCG cells achieving clear discrimination from background noise (non-cellular particles). Moreover, adapting reference fluorogenic beads that have a known quantity during the flow cytometric analysis turned this technique into a rapid quantitative assay for better identification, sorting, and quantification of various types of cells in a short time with minimal human intervention^22–26^. It also eliminates the need to couple the flow cytometer to the Coulter counter for counting the whole population, hence minimizing the need for additional apparatus and sample processing.

The ability of the flow cytometer with TO/PI dyes to distinguish the three forms of BCG cells (dead, injured and live) was noteworthy. Injured cells probably can exclude PI dye or uptake FD dye to some extent and hence can be miscounted among dead and living cells leading to inaccuracy when a single stain is used^14,20^. The present study also demonstrated that the injured cells are not living cells, as evidenced by culturing heat-treated cells on LJ medium. Yet, the sensitivity of the flow cytometer can be further increased by increasing the number of events acquired for each sample. According to Domingo et al.^38^, at least tenfold more cells must be acquired to increase sensitivity by 1-log^38^.

Verification of validation parameters for flow cytometry showed an acceptable intra- and inter-assay precision. Also, the data obtained using this technique showed a perfect positive correlation with the CFU assay.

In previous studies, the expensive Middlebrook 7H9 broth was the preferred medium for reconstituting and diluting the BCG vaccine for potency testing^14,15,18^. In the present study, Sauton’s solution containing per liter: 0.5 g ferric ammonium citrate, 3 g l-asparagine, 0.5 g MgSO_4_.7H_2_O, 2 g potassium phosphate, 1 g ZnSO_4_, 50 ml glycerol and supplemented with 10% v/v tween 80 (pH 7.3) was evaluated for reconstitution and dilution of Mycobacteria.

Although the statistical analysis showed no significant difference between the results obtained from the CFU assay method and our proposed approaches in terms of the BCG vaccine viability, the results of the P-value and Pearson correlation for the flow cytometry were overall better than that of the colorimetric assay. Supplementary Fig. S2 presents a comparison of the percentage difference between the estimated viable cell concentration of 10 BCG vaccine batches as determined by the colorimetric MTT assay and flow cytometric analysis from that of the official CFU assay. The box plot shows the maximum, minimum, mean, and median of the % difference, besides the limit for the first and third quartiles. The percentage differences for flow cytometry didn’t exceed 5%, whereas for the MTT assay, 4 out of 10 samples had differences greater than 5%. This provides an additional reason to consider the flow cytometry assay as a more favorable technique for BCG vaccine potency testing than the colorimetric assay.

An additional limitation of the MTT assay is that it provides a relative rather than an absolute count of the living cells. In the case of flow cytometric analysis, this will only be true if no reference beads were included in the assay since the machine will provide information on the percentage of the different cells in the whole population. To get the absolute count, an external counting machine (Coulter counter) should be used. However, the introduction of the reference counting beads turns the flow cytometer into an absolute counter^39,40^.

In conclusion, vaccine manufacturing is a bulk, rapidly developing industry that has witnessed extensive growth within the last few decades. The potency test is a crucial quality control parameter of live-attenuated vaccines. Several automated techniques were developed to overcome the limitations of the golden official colony-forming unit assay of bacterial vaccines. Flow cytometry technique offers several advantages over culture-based and biochemical techniques. It has superior outcomes with regard to assay time, sample processing, amount of generated data, sorting and discrimination power, among others. However, proper validation is always demanded. Once validated, this technique can be employed effectively to vaccine industrially, hence, saving time, increasing precision and accuracy, and lowering the analysis cost.

## Methods

### Chemicals and culture media

Lyophilized BCG vaccine lots sourced from the Serum Institute of India (Russian BCG-I) with 2–8 × 10^6^ CFU/ml were used. Sauton’s solution supplemented with 10% Tween 80 and Lowenstein-Jensen media were provided by VACSERA (Giza, Egypt). BD Cell Viability Kit of thiazole orange dye (TO)/propidium iodide dye (PI) and BD liquid counting beads were products of Becton, Dickinson and Company (New Jersey, USA). Phosphate-buffered saline (PBS), tween 20, tween 80, ethylenediaminetetraacetic acid (EDTA), and 3-(4,5-dimethylthiazol-2-yl)-2,5-diphenyltetrazolium bromide (MTT) were obtained from Sigma Aldrich (Massachusetts, USA), while, Dimethyl sulfoxide (DMSO) was obtained from Thermo Fisher Scientific (Massachusetts, USA).

### Determination of viable cell count of BCG vaccine by CFU assay

The potency test of BCG vaccine batches was assessed through CFU assay as described elsewhere^10,41^. Briefly, the dried lyophilized BCG vaccine was reconstituted using 1 ml Sauton’s solution—10% Tween 80. Three successive twofold dilutions of the reconstituted vaccine were prepared 1/5000, 1/10,000, and 1/20,000 using Sauton’s solution—10% Tween 80. Then 100 μl of each dilution was inoculated in tubes of LJ media. The inoculated media were incubated at 37 °C for 4 weeks. In the first three days, the tubes were incubated horizontally, all tubes were swirled gently to ensure homogenous distribution of the diluted vaccine, and then the tubes were maintained in an upright position till the end of the incubation period. After four weeks, the observed colonies were counted.

### Colorimetric assay

#### Determination of viable cell count of BCG vaccine by colorimetric assay

The colorimetric assay of the BCG vaccine was done as described earlier^15,42,43^. Ten BCG lots and a reference BCG vaccine were reconstituted in 1 ml Sauton’s solution—10% Tween 80, respectively. The reference BCG vaccine was used to generate a calibration curve by initially generating two-fold serial dilutions in a microtiter plate. A hundred microliters of each concentration of the reference cell suspension and the test samples were transferred to a 96-well plate. Twenty microliters of MTT solution (5 mg of salt in 1 ml PBS) were then added to each well including the blank. The plate was incubated at 37 °C for 48 h in a standing incubator (Heraeus, USA). At the end of the assay, 100 μl of DMSO was added to each well to dissolve the formed formazan. The optical density of each well was measured at 570 nm using microtiter plate reader (VICTOR™ X Multilabel Plate Reader, PerkinElmer, Germany). The experiment was performed in three independent replicates.

#### Assessment of validation parameters of the colorimetric assay for determination of viable cells of BCG vaccine

The analytical validation method aims to ensure that the data were credible and reproducible. Therefore, this validation protocol includes verification of precision (inter- and intra), accuracy, linearity, range, and limit of detection^29,30,44,45^.

##### Linearity

Linearity assay tests the ability to obtain test results directly proportional to the analyte concentration in the sample within a given range. It was calculated from the three calibration curves performed for three consecutive days.

##### Precision

The intra-assay precision was performed by running ten measurements within the same day and then the mean, standard deviation (SD) and percentage coefficient of variation (CV%) Eq. (1) of each concentration were calculated using the calibration curve equation. Finally, the mean of CV% for all the measurement (intra-assay CV%) was obtained.

For measuring the inter-assay precision, four samples were repeatedly measured over three consecutive days. A calibration curve was attained for each day. The BCG viability in each sample was calculated using the calibration curve equation and the mean of each sample and day were obtained. Subsequently, the mean of the means, SD of the means, and CV% of the means was determined and the average CV% was considered as inter-assay CV%.

1 $$CV\% = \frac{{SD}}{{Mean}} \times 100.$$

##### Accuracy

The accuracy parameter was performed using five BCG vaccine reference standards and was verified to express the percentage error between the observed value and the expected value using Eq. (2):

2 $$\% \,Error = \frac{{\left| {Observed - Expected\left. {} \right|} \right.}}{Expected} \times 100.$$

##### Limits of detection (LOD)

LOD is the lowest amount of analyte in the sample that can be detected. LOD was calculated using Eq. (3).

3 $$LOD = \frac{{3.3 \times {\text{standard }}\,{\text{deviation of response}}}}{{{\text{slope}}\,\,{\text{of}}\,{\text{the}}\,\,{\text{calibration}}\,{\text{curve}}}}.$$

### The flow cytometry assay

#### Sample preparation and flow cytometry analysis

Ten lots of BCG vaccine were analyzed using a flow cytometer with TO/PI dye kit combined with counting fluorogenic beads. The assay was initiated by reconstituting all the samples in 1 ml Sauton’s solution – 10% Tween 80, and incubating at 37 °C for 24 h. The resulting suspension was diluted (1:100) in the staining buffer (freshly prepared PBS containing 0.01% Tween 20 and 1 mmol/l EDTA)^39,46^. For analysis, 200 µl of the samples were mixed with 5 µl each of PI and TO dyes and the mixtures were incubated for 5 min at room temperature and then analyzed by flow cytometer. Fifty thousand events were acquired for each sample.

#### The ability of flow cytometry assay to distinguish between live and dead cells

Two BCG vaccine batches were reconstituted and cultured in 1 ml Sauton’s solution—10% Tween 80 and incubated at 37 °C for 24 h. The BCG cells in the two batches were killed, one by autoclaving (STERIS®, USA) at 121 °C for 30 min, while the other was treated with 2.5% formaldehyde. Subsequently, 200 µl of the treated samples were mixed with 5 µl each of PI and TO dyes and the mixtures were incubated for 5 min at room temperature and then analyzed by flow cytometer. The same batches of treated BCG vaccine were cultured on LJ media and incubated at 37 °C for 28 days as a control^47,48^.

#### Determination of viable cell count of BCG vaccine using flow cytometry technique

Ten batches of BCG vaccines were diluted in PBS (1:100) and incubated at 37 °C for 24 h. Each batch’s live-cell count was measured by flow cytometry (BD FACSCanto™ II, BD Life Sciences, CA, USA) equipped with 488 nm-laser excitation and BD CellQuest™ software package (BD Life Sciences, CA, USA). Side scatter (SSC) was used for microbial cells. Overall, 50,000 events were acquired for each sample. Combined parameter of FSC-H, SSC-H and FL2 was used to gate the cells and the counting beads, and then sequential gating was done using FL1 *vs.* FL3 dot plots of bacteria stained with TO and PI. TO fluoresce primarily in FL1 and FL2; PI fluoresces primarily in FL3. BD FACSDiva Software was used for data acquisition, data analysis and visualization (BD Life Sciences, CA, USA). Equation (4) was used to calculate the absolute count of viable cells in the sample:

4 $$Concentration\,\,\,of\,\,\,bacterial\,\,population = {\raise0.7ex\hbox{${N1}$} \!\mathord{\left/ {\vphantom {{N1} {N2}}}\right.\kern-0pt} \!\lower0.7ex\hbox{${N2}$}} \times {\raise0.7ex\hbox{${N3}$} \!\mathord{\left/ {\vphantom {{N3} V}}\right.\kern-0pt} \!\lower0.7ex\hbox{$V$}} \times D,$$

where N1 is the number of events in a region containing cell population, N2 is the number of events in bead population, N3 is the number of beads per test (as obtained from the BD Liquid Counting Beads label), D is the dilution factor and V is the test volume.

#### Assessment of validation parameters for flow cytometry

##### Precision

Ten BCG vaccine batches were analyzed by flow cytometry, and each sample was repeated three times sequentially. On the other hand, inter-precision has been performed by three-time analysis of four BCG vaccine batches on three consecutive days.

### Statistical analysis

All experiments were performed in triplicates except for the CFU assay which was run in duplicate. The presented data represent the mean of these replicates ± standard deviation. The viability of the BCG lots performed using the colorimetric assay was done by substituting the calibration curve equation. The Microsoft Excel Analysis ToolPak Add-in was used for performing Student’s *t* test and Pearson correlation to compare the results of the analysis of BCG batches using MTT and flow cytometry assays to the standard CFU assay.

## Supplementary Information

Supplementary Information.

## Data Availability

The datasets generated during and/or analyzed during the current study are available from the corresponding author on reasonable request.
