# Supplementary material for: Development of a fast and precise potency test for BCG vaccine viability using flow cytometry compared to MTT and colony-forming unit assays
Source: Sci Rep. 2023 Jul 18;13:11606. doi: 10.1038/s41598-023-38657-x (PMC10354009; doi:10.1038/s41598-023-38657-x)
Supplement: Supplementary file 1 — Supplementary Information. [file 41598_2023_38657_MOESM1_ESM.docx]

Development of a fast and precise potency test for BCG vaccine viability using flow cytometry compared to MTT and colony-forming unit assays

*Hend M. Moghawry ^a,b^, Mohamed E. Rashed ^b^, Kareeman Gomaa ^c^, Sameh AbdelGhani ^a,d^, Tarek Dishisha^a,^**

*^a^ Department of Pharmaceutical Microbiology and Immunology, Faculty of Pharmacy, Beni-Suef University, 625 11 Beni-Suef, Egypt.*

*^b^ General Administration of Biological Products, Central Administration of Biological and Innovative Products and Clinical Trials, Egyptian Drug Authority (EDA), Egypt*

*^c^ Clinical and Chemical Pathology Department, Faculty of Medicine - Kasr Al-Ainy, Cairo University, Cairo, Egypt.*

*^d^ Department of Pharmacy, Jewish Hospital, University of Louisville, Louisville, KY – 402 02 Kentucky, USA*

* **Corresponding author:**

E-mail: Tarek.Dishisha@Pharm.bsu.edu.eg

Phone: +20-82-2162133

Fax: +20-82-2162133


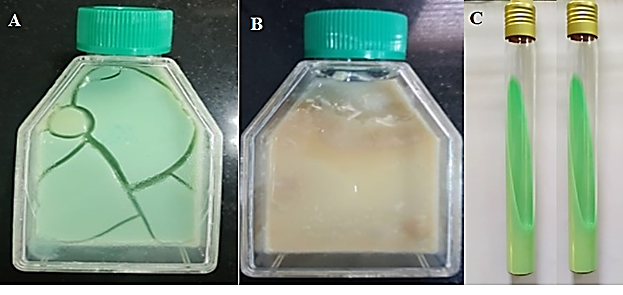


**Supplementary Fig. S1. (A), (B) Cracking of the Lowenstein-Jensen media during cultivation of mycobacterial cells showing media cracking. (C) Culture of killed BCG vaccine on Lowenstein-Jensen media showing no colonies. Photos were taken by: Hend M. Moghawry.**

**
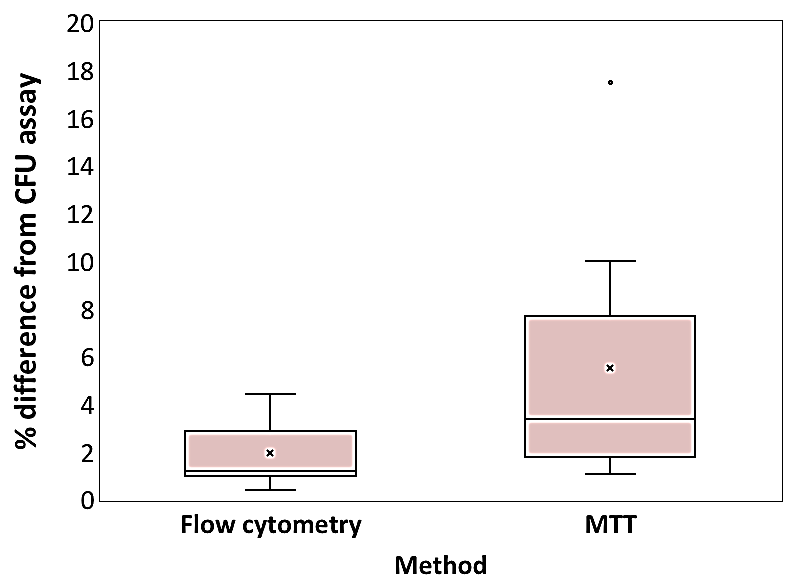
**

**Supplementary Fig. S2. Box plot of the percentage difference between the official colony-forming unit assay (CFU) and the flow cytometry assay or the colorimetric MTT assay. The percentage difference was calculated for 10 BCG vaccine batches. The estimated viable cell concentration was determined using standard BCG calibration curve in case of the colorimetric MTT assay, while, using BD Cell Viability Kit with thiazole orange (TO) /propidium iodide (PI) dyes and liquid counting beads in case of flow cytometry.**

**
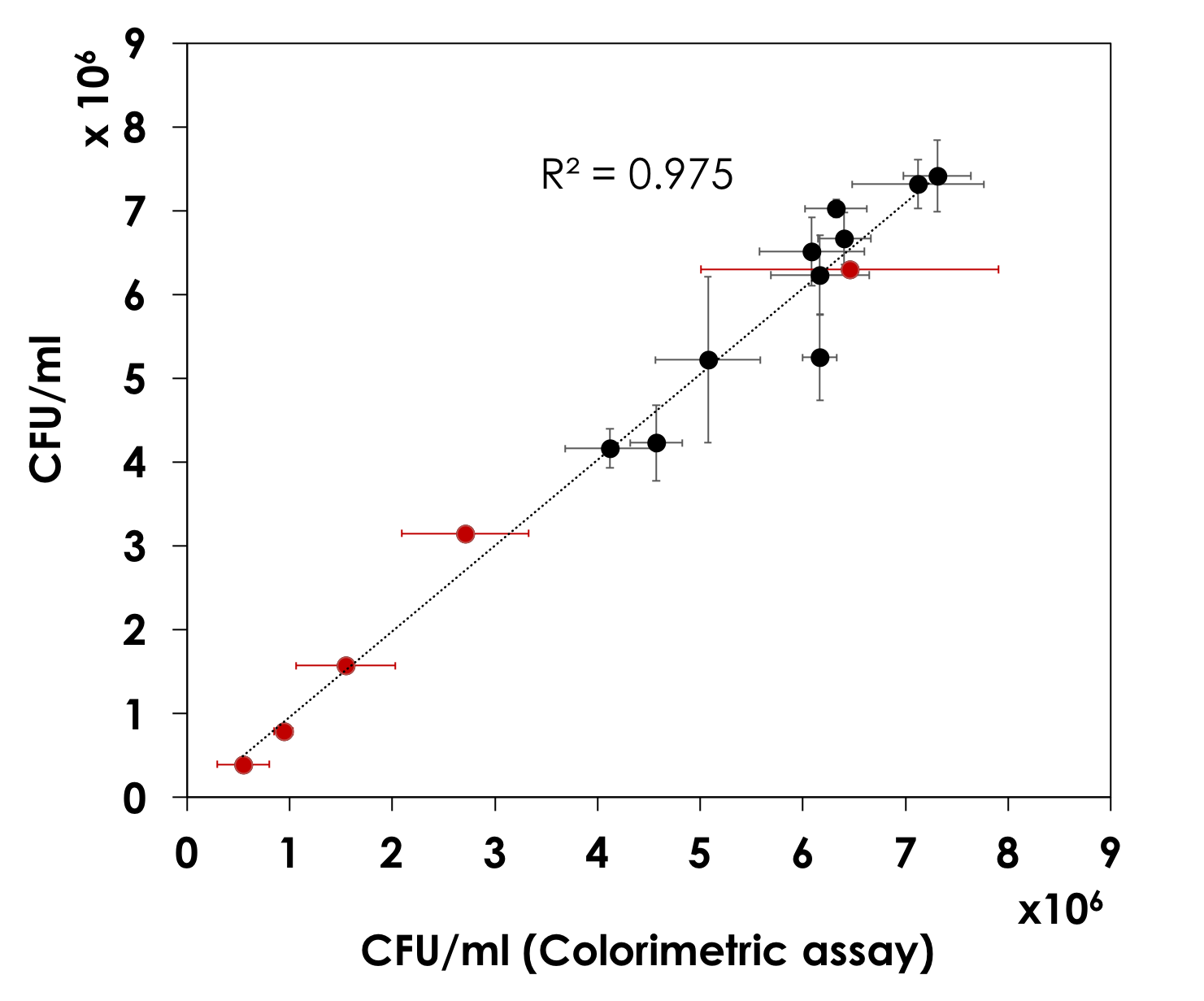
**

**Supplementary Fig. S3. Correlation of the viability of Bacille Calmette-Guérin (BCG) vaccine as determined by the official colony-forming unit (CFU) assay and colorimetric MTT assay using 10 sample batches (Black Circles) and 5 standard batches (Red Circles).**

**Supplementary Table S1. Intra-assay precision analysis of the colorimetric MTT assay considering the results of 10 BCG vaccine batches analyzed in triplicates**

| **Batch No.** | **Estimated BCG viability** | | | **Parameters** | | |
| --- | --- | --- | --- | --- | --- | --- |
|  | **Read 1*** | **Read 2*** | **Read 3*** | **Mean*** | **SD*^,a^** | **CV%** |
| **A** | 4.86 | 4.70 | 5.66 | 5.08 | 0.51 | 10.09 |
| **B** | 7.57 | 6.94 | 7.41 | 7.31 | 0.33 | 4.54 |
| **C** | 4.47 | 4.39 | 4.86 | 4.57 | 0.26 | 5.60 |
| **D** | 4.62 | 3.91 | 3.83 | 4.12 | 0.44 | 10.65 |
| **E** | 5.50 | 6.46 | 6.30 | 6.09 | 0.51 | 8.42 |
| **F** | 6.62 | 6.22 | 5.66 | 6.17 | 0.48 | 7.79 |
| **G** | 6.54 | 7.81 | 7.02 | 7.12 | 0.64 | 9.04 |
| **H** | 6.46 | 5.98 | 6.54 | 6.32 | 0.30 | 4.77 |
| **I** | 6.22 | 6.70 | 6.30 | 6.40 | 0.26 | 4.00 |
| **J** | 5.98 | 6.30 | 6.22 | 6.17 | 0.17 | 2.69 |
| **Intra-assay CV% = 6.76%** | | | | | | |
| * (x 10^6^ CFU/ml) ^a^ SD = Standard Deviation | | | | | | |

**Supplementary Table S2. Correlation of viability of BCG vaccine as determined by the official colony-forming unit (CFU) assay and colorimetric MTT assay**

| **Batch No.** | **CFU assay*** | **Colorimetric assay*** |
| --- | --- | --- |
|  | **Mean of triplicate analysis** | **Mean of triplicate analysis** |
| **A** | 5.22 | 5.08 |
| **B** | 7.42 | 7.31 |
| **C** | 4.23 | 4.57 |
| **D** | 4.16 | 4.12 |
| **E** | 6.51 | 6.09 |
| **F** | 6.23 | 6.17 |
| **G** | 7.32 | 7.12 |
| **I** | 7.03 | 6.32 |
| **J** | 6.67 | 6.40 |
| **K** | 5.25 | 6.17 |
| * (x10^6^ CFU/ml) | | |

**Supplementary Table S3. Intra-assay precision analysis of the flow cytometry technique for analysis of 10 BCG vaccine samples using BD Viability Kit with thiazole orange (TO) /propidium iodide (PI) dyes and liquid counting beads**

| **Batch No.** | **Estimated BCG viability** | | | **Parameters** | | |
| --- | --- | --- | --- | --- | --- | --- |
|  | **Read 1*** | **Read 2*** | **Read 3*** | **Mean*** | **SD*** | **CV%** |
| **A** | 7.55 | 7.20 | 7.36 | 7.37 | 0.18 | 2.39 |
| **B** | 4.01 | 3.40 | 3.88 | 3.76 | 0.32 | 8.57 |
| **C** | 3.89 | 4.19 | 4.13 | 4.07 | 0.16 | 4.00 |
| **D** | 4.23 | 4.45 | 4.59 | 4.42 | 0.18 | 4.12 |
| **E** | 3.39 | 3.09 | 3.67 | 3.39 | 0.29 | 8.66 |
| **F** | 4.80 | 4.50 | 4.89 | 4.73 | 0.20 | 4.30 |
| **G** | 3.85 | 3.35 | 4.00 | 3.74 | 0.34 | 9.09 |
| **H** | 5.46 | 5.12 | 5.87 | 5.48 | 0.37 | 6.81 |
| **I** | 3.76 | 3.22 | 3.10 | 3.36 | 0.3.5 | 10.45 |
| **J** | 4.86 | 5.09 | 5.05 | 5.00 | 0.12 | 2.41 |
| **Intra-assay CV% = 6.08%** | | | | | | |
| * (x 10^6^ CFU/ml) | | | | | | |

**Supplementary Table S4. Correlation of viability of BCG vaccine as determined by the official colony-forming unit (CFU) assay and the flow cytometric assay using BD Viability Kit with thiazole orange (TO) /propidium iodide (PI) dyes and liquid counting beads**

| **Batch No.** | **Flow cytometry assay*** | **CFU assay*** |
| --- | --- | --- |
|  | **Mean of triplicate analysis** | **Mean of triplicate analysis** |
| **A** | 7.37 | 7.44 |
| **B** | 3.76 | 3.78 |
| **C** | 4.07 | 4.02 |
| **D** | 4.42 | 4.47 |
| **E** | 3.39 | 3.28 |
| **F** | 4.73 | 4.68 |
| **G** | 3.74 | 3.66 |
| **H** | 5.48 | 5.27 |
| **I** | 3.36 | 3.52 |
| **J** | 5.00 | 5.04 |
| * (x10^6^ CFU/ml) | | |

**Supplementary Table S5. Intra-assay precision analysis of the colony-forming unit (CFU) technique for analysis of 20 BCG vaccine samples using Lowenstein-Jensen media.**

| **Batch No.** | **BCG viability** | | **Parameters** | | |
| --- | --- | --- | --- | --- | --- |
|  | **Read 1*** | **Read 2*** | **Mean*** | **SD*** | **CV%** |
| A1 | 5.92 | 4.52 | 5.22 | 0.99 | 18.95 |
| B1 | 7.11 | 7.72 | 7.42 | 0.43 | 5.79 |
| C1 | 3.91 | 4.55 | 4.23 | 0.45 | 10.70 |
| D1 | 4.33 | 4.00 | 4.16 | 0.23 | 5.62 |
| E1 | 6.23 | 6.80 | 6.51 | 0.40 | 6.20 |
| F1 | 5.90 | 6.57 | 6.23 | 0.47 | 7.60 |
| G1 | 7.52 | 7.11 | 7.32 | 0.29 | 3.96 |
| H1 | 6.95 | 7.10 | 7.03 | 0.11 | 1.50 |
| I1 | 6.45 | 6.89 | 6.67 | 0.31 | 4.68 |
| J1 | 4.89 | 5.61 | 5.25 | 0.51 | 9.78 |
| A2 | 7.90 | 6.99 | 7.44 | 0.65 | 8.67 |
| B2 | 3.55 | 4.01 | 3.78 | 0.33 | 8.69 |
| C2 | 4.26 | 3.79 | 4.02 | 0.33 | 8.28 |
| D2 | 4.89 | 4.05 | 4.47 | 0.59 | 13.29 |
| E2 | 3.44 | 3.13 | 3.28 | 0.22 | 6.61 |
| F2 | 5.01 | 4.35 | 4.68 | 0.47 | 9.97 |
| G2 | 3.73 | 3.58 | 3.66 | 0.11 | 2.91 |
| H2 | 5.57 | 4.98 | 5.27 | 0.42 | 7.94 |
| I2 | 3.75 | 3.28 | 3.52 | 0.33 | 9.47 |
| J2 | 5.20 | 4.89 | 5.04 | 0.22 | 4.39 |
| **Intra-assay CV%** | | | | | **7.75%** |
| * (x10^6^ CFU/ml) | | | | |  |
